# Resident physician burnout and association with working conditions, psychiatric determinants, and medical errors: A cross-sectional study

**DOI:** 10.1371/journal.pone.0312839

**Published:** 2024-10-30

**Authors:** Vithawat Surawattanasakul, Penprapa Siviroj, Wuttipat Kiratipaisarl

**Affiliations:** 1 Department of Community Medicine, Faculty of Medicine, Chiang Mai University, Chiang Mai, Thailand; 2 Environmental Medicine and Occupational Medicine Excellent Center, Faculty of Medicine, Chiang Mai University, Chiang Mai, Thailand; Zagazig University Faculty of Human Medicine, EGYPT

## Abstract

Burnout has become a significant occupational concern for resident physicians, primarily attributed to chronic workplace stressors, inadequate work-life balance, high expectation from attending staffs, steep learning curve, and limited patient care experience. The study aimed to investigate the prevalence and associated factors of burnout in medical residents. This cross-sectional study was conducted online questionnaire of all specialists in a university hospital from September to October 2022. Burnout was assessed using the Maslach Burnout Inventory-Human Services Survey for Medical Personnel. The data collection encompassed information on socio-demographics, working conditions, psychiatric issues, and medical errors as potential predictive variables. To analyze the association between these factors and burnout, a confounder summary score model was employed in four separate models utilizing multivariable logistic regression. A total of 238 participants, the average age of participants was 28.1 years (SD 2.7), and 56.2% of them were female. Weekly working hours averaged 75 (SD 21.8). Burnout prevalence was 46.3%. This prevalence was characterized by high levels of emotional exhaustion (57.1%) and depersonalization (36.1%), along with low levels of personal accomplishment (52.4%). Summary of association in each domain with burnout were as follow: demographic determinants, (adjusted odds ratio (aOR) 2.80, 95% CI 1.68–4.64), working conditions (aOR 2.97, 95% CI 1.54–5.71), psychiatric determinants (aOR 2.47, 95% CI 1.77–3.45) medical errors (aOR 2.14, 95% CI 1.05–4.34). Medical residency training programs should provide a supportive system that actively monitors and addresses depressive symptoms. Implementing preventive measures, such as increasing pay rates, can play a role in mitigating burnout.

## Introduction

Burnout syndrome has emerged as an occupational phenomenon resulting from prolonged exposure to psychosocial stress in the workplace [1, 2]. While not officially categorized as a medical disease, burnout syndrome had several potentially negative impacts on workers’ performance in both industrial and service sectors [3–5]. It manifests through indicators such as early resignation, suicidal ideation, and adverse health effects [6], all contributing to diminished organizational efficiency [5, 6]. Unfortunately, occupation in the human service sector, particularly the medical profession, bears a significant burden of burnout, especially in the earlier career stages [4, 7]. Globally, as investigated in the latest meta-analysis of prevalence, burnout was estimated to be 51% (95%CI, 45–57%), with a higher proportion in certain region (Asia and the United States), and certain specialties particularly in radiology (77.2%), neurology (72%) and surgery (58.4%). Previous studies conducted in Thailand consistently reveal a high prevalence of burnout during residency training. This trend extends to burnout subdomains, with reported prevalence rates of high emotional exhaustion (EE), high depersonalization (DP), and low personal accomplishment (PA) ranging from 17.0% to 53.8%, 12% to 45.5%, and 0% to 94.3%, respectively, across various study [8–15].

Burnout among residents are well-acknowledged challenges in graduate medical education. The nature of resident training can contribute to a substantial level of burnout, impacting an individual’s ability to navigate diagnostic dilemmas and engage in complex treatment decision-making. Residency training stands out as one of these critical stages requiring significant adjustment due to the additional occupational stress from the combination of high job demand and low job control. Residency training varies widely across countries, with differences in duration, structure, and specialization options. In Thailand, the residency training is typically aligned with the United States Association of Graduate Medical Education (ACGME) which consist of three to five years of training. University hospitals, are the most common setting in residency training activities, in which the population consist of a mixture of physicians who continued their postgraduate education from the university hospital itself and residents who transition from the other public hospitals for their residency training [16–18].

Evidence suggests that the factors contributing to burnout syndrome in medical residency training encompass two major domains: sociodemographic factors, such as female gender [14], younger age [14], working conditions, such as difficult relationships with coworkers [11], and psycho-social factors, such as thoughts of resignation [11]. However, when considering each domain of burnout, both unique and shared determinants emerge. Shared risk factors across all three domains can be grouped into two main categories: poor working conditions and personal psycho-social conditions. Poor working conditions include high workload [8–10], suboptimal work environment [12, 15], and strained co-worker relationship [8, 12, 13, 15]. Personal psycho-social conditions include elevated family burden, inadequate sleep, and reactive coping mechanism [9]. Unique risk factors that present specific to low PA include sociodemographic factor, such as being male [8] and attitudinal factor, such as negative perception to medical practice [9]. Nevertheless, an aspect that remains inconsistent is the role of medical errors, which studies showing findings regarding their association with burnout [19].

The gaps of knowledge existed in Thai resident population consisted of heterogeneity in each training center curriculum, and exposure characteristic regarding psycho-social factors, which required exploration that incorporate center-specific evidence in order to manage the problem effectively in each training center. Moreover, most of existing studies focus on attending specialists in single specialty rather than the broader resident population, making it challenging to apply the findings to improve the overall curriculum. Recognizing this gap, this study aimed to investigate the prevalence and determinants of burnout syndrome among broad domain consisting of all resident physicians training in all specialties in a tertiary-care university hospital in Thailand.

## Materials and methods

### Study design, setting, and participants

This cross-sectional was conducted as an online survey-based investigation in a university hospital in northern Thailand throughout the academic year 2022, spanning from 1 September to 31 October. The participants included formal in-training resident physicians of all specialists within this hospital. The inclusion criteria required participants to be Thai physicians working in this university hospital for a minimum of 2 months on the day they completed the questionnaire. Exclusions were made for physicians on elective rotations outside the hospital, as well as those who were not formally registered into the curriculum in the current semester. The initial study population comprised 519 individuals. To determine the sample size (N), Cochran’s formula, $N=\frac{Z^{2}p(1-p)}{d^{2}}$, was employed, considering a prevalence (p) of 10.74% [13], an absolute error of 0.04 (d), a 95% confidence interval (z-statistic 1.96), and a pre-specified design effect of 1.0, resulting in a sample size of 231.

Invitation forms were distributed to physicians via the department’s official channels and authors during departmental meetings or academic activities for the invite to join the project by clarifying the project objectives, and duration to answer the questionnaire, and showing a QR code or link for entering the online questionnaire.

**Fig 1:**
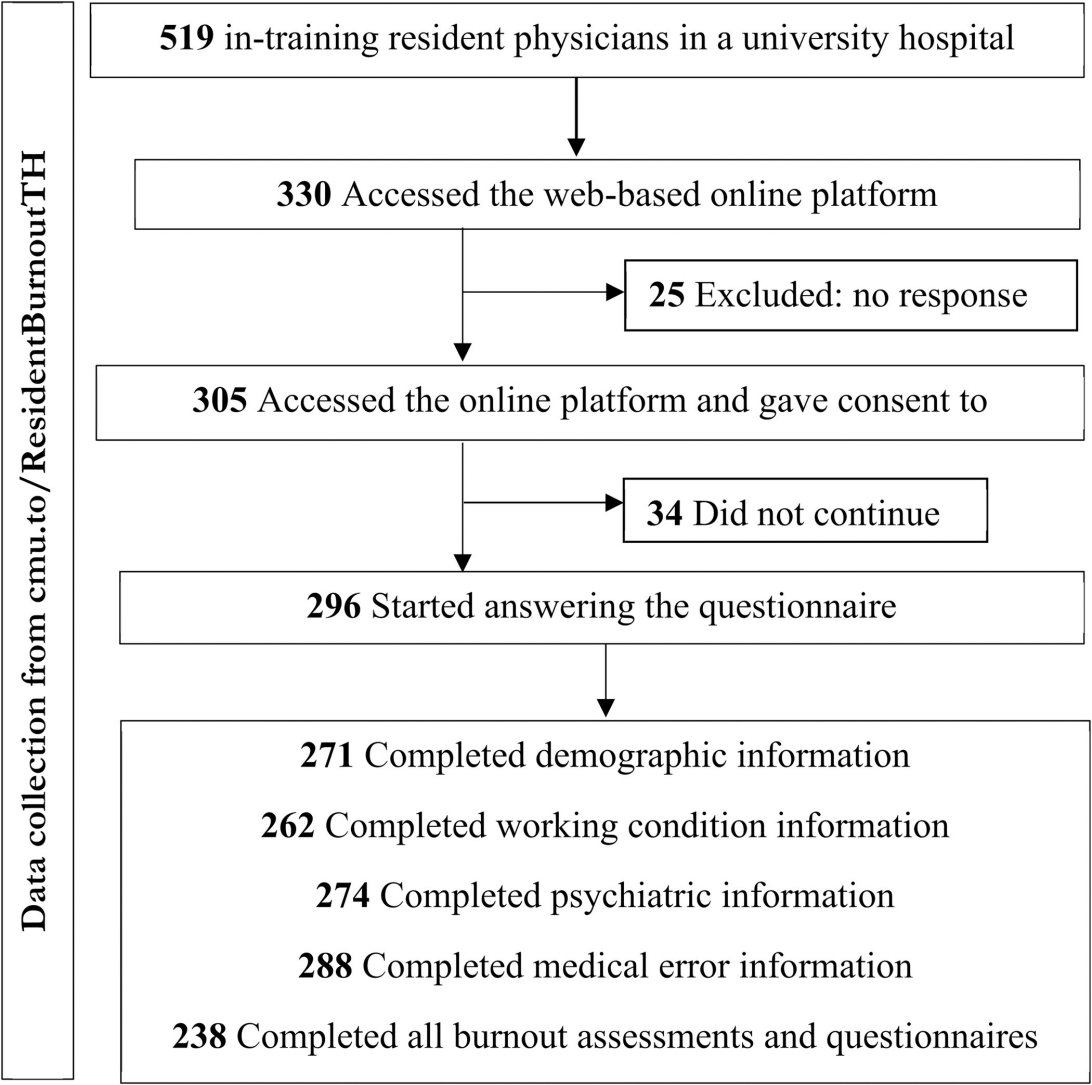
Study flow diagram of participant recruitment.

The study flow, illustrated in Fig 1, outlines the participation process for 519 in-training resident physicians. Among this group, 305 participants engaged in the questionnaire. Notably, 238 participants, constituting 45.9% of the total, successfully completed the entire questionnaire.

### Measurement

The data were gathered through a self-administered online questionnaire consisting of two main parts: the outcome variable, focusing on the assessment of burnout syndrome, and determinants such as socio-demographics, working conditions, psychiatric issues, and medical errors. This survey was facilitated using an online questionnaire platform called Research Electronic Data Capture (REDCap), conducted in the Thai language, and hosted by Chiang Mai University [20, 21].

#### Outcome variable

Burnout syndrome was assessed in all participants using the 22-item Maslach Burnout Inventory. Each item was evaluated on a 7-point Likert scale based on frequency, ranging from 0 to 6. The rating scores for the EE and PA subdomains were designed as follows: “0 = Never, 1 = A few times a year or less, 2 = Once a month or less, 3 = A few times a month, 4 = Once a week, 5 = A few times a week, and 6 = Every day”. Notably, the PA subdomain had an inverse rating score. The burnout questionnaire was structured into three subdomains, comprising 9 questions on EE, 5 questions on DP, and 8 questions on PA. Burnout classification and subdomain cut points recommended by the MBI manual, third edition, were utilized [2]. EE levels were categorized as low (0–18), moderate (19–26), and high (≥27). DP levels were categorized as low (0–5), moderate (6–9), and high (≥10). PA levels were delineated as high (≥ 40), moderate (34–39) scores, and low (0–33) [1, 2]. This study reported Cronbach’s alpha values for the EE subdomain as 0.946, the DP subdomain as 0.838, and the PA subdomain as 0.876 [2, 22, 23]. High scores in EE and DP, along with low scores in PA, were indicative of burnout.

#### Determinant variables

The determinant variables encompassed four primary domains based on their contextual nature: socio-demographics, working conditions, psychiatric issues, and medical errors.

The first part, focusing on demographic determinants, included variables such as gender (male or female), age (in years), marital status (single, married), physical underlying disease (present or absent), smoking status (active, non-active, or never), alcohol drinking status (active, non-active, or never), and exercise frequency (days per week).

The second part, centered on working conditions determinants, comprised factors such as year of residency training (first to six years), original affiliation (Ministry of Public Health, Ministry of Tertiary Education), departments (major departments–surgery, internal medicine, obstetrics and gynecology, pediatrics, and orthopedics–or other departments), total work hours per week, monthly salary (in THB), and financial status (just enough, more than enough, or inadequate).

The third part, psychiatric determinants, focused on participant-reported variables such as active psychiatric underlying disease (yes or no), suicidal ideation, and depressive symptoms [5]. It incorporated the measurement of psychiatric determinants, including sleep duration (average hours per day over the last month). Suicidal ideation was assessed through a single-item measurement of suicidal behaviors, where participants indicated whether they had experienced suicidal thoughts in the past 12 months with a yes or no response [5, 24]. Depressive symptoms were examined using the nine-item PHQ-9 instrument, a widely accepted tool for screening and diagnosing Major Depressive Disorder (MDD). Participants rated the severity of their symptoms on a 4-point Likert scale PHQ-9, with depression severity classified based on the suggested optimal cutoff point in the Thai population for the screening of clinical depression according to the Thai-translated version by Panyawong W. [25]: mild depressive symptoms (0–8) and moderate to high depressive symptoms (9–27). The Cronbach’s alpha for this instrument was 0.79.

The fourth part involved self-reporting medical errors using a 4-item questionnaire, including common medical errors in clinical settings such as severe, non-severe, medication prescription, and laboratory investigation errors. The frequency of each type of error in the last 3 months was assessed as either at least one or none [5, 26].

### Statistical analysis

All statistical analyses were carried out using the STATA version 16.0 package (Stata Corp. 2019. Stata Statistical Software: Release 16, College Station, TX, USA: Stata Corp LLC.). Descriptive statistics, including percentages for categorical variables and means with standard deviations for continuous variables, were employed to characterize the data. Normality was explored using visualization with histogram and quantile-quantile plots. The prevalence of burnout in each domain, EE, DP, and PA, was determined by dividing the counts of high EE, high DP, and low PA by the total number of participants. Factors associated with burnout and each domain were analyzed separately using exploratory multivariable binary logistic regression models, with high EE, high DP, and low PA as outcome variables. Given the potential for a high number of determinant variables compared to the sample size, which could be unsuitable for regression analyses, with potentially fewer than 10 burnout cases per variable with 24 degrees of freedom [27–29], the investigators chose the confounder summary score concept [30, 31]. This approach involved grouping determinant variables into four separate multivariable binary logistic regression models, according to the contextual nature of the determinants. The linear predicted values from the logit function in each model were fitted into the final model, providing the summary of how each domain influenced the others. Adjusted odds ratio and 95% confidence intervals were used to describe the association between burnout and each domain and its associated factors. The outlier was prevented by limiting the range of continuous determinant variables into a plausible range. Additional measures on outlier and heteroskedasticity were managed with the usage of Huber-White robust variance correction method in the statistical modeling steps. Upon the assumption of missing-at-random mechanism (MAR), multiple imputation was used for handling of missing data. The imputation was carried out using the chained multivariate regression. The process used 25 preliminary burn-in imputations, followed by 25 multiple imputations. The set of determinants of substantive interest in each of the four multivariable binary logistic regression models were estimated in each imputed dataset separately. Rubin’s rule was applied for the pooling of standard errors. A complete case analysis was carried out as a supplement to compare the similarities and differences of the results. Two-tailed significance levels of 0.05 were considered in the statistical analyses. The recruitment process and study results were reported following the STROBE (Strengthening the Reporting of Observational Studies in Epidemiology) guidelines [32].

### Ethical considerations

This study was conducted following the Declaration of Helsinki guidelines and the protocol was approved by the Research Ethics Committee of the Faculty of Medicine, Chiang Mai University, Thailand (079/2022, date of approval 24 May 2022).

## Results

### Baseline characteristics of the participants

The study involved the distribution of 519 questionnaires, resulting in 296 partial responses and 238 completed responses, translating into the response rate of 45.9%. Missing data in partial response were most prevalent in salary with 21 (7.1%) responses missing. Table 1 provides a summary of the baseline characteristics of participants. The average age was 28.1 years (SD ±2.7), and gender distribution was 56.2% females and 43.8% males. A majority identified as non-smokers (96.2%), with 27.6% being active drinkers. Most participants were single (92.5%), while 13.3% reported financial inadequacy.

**Table 1:** Participant baseline characteristics.

	Number (%), mean ±SD			Missing (%)	*p*-Value
Determinants	Total	Burnout	Not burnout		
	(*n* = 296)	(*n* = 137, 46.3%)	(*n* = 159, 53.7%)		
**Gender**					
Male	128 (43.8)	71 (53.0)	57 (36.1)	4 (1.4)	**0.004**
Female	164 (56.2)	63 (47.0)	101 (63.9)		
**Age (years)** (*n* = 293)	28.1 ±2.7	27.9 ±2.3	28.3 ±2.9	3 (1.0)	0.24
**Physical underlying disease**					
No	206 (70.3)	91 (67.4)	115 (72.8)	3 (1.0)	0.37
Yes	87 (29.7)	44 (32.6)	43 (27.2)		
**Smoking status**					
Never or non-active smoker	282 (96.2)	125 (92.6)	157 (99.4)	3 (1.0)	**0.003**
Active smoker	11 (3.8)	10 (7.4)	1 (0.6)		
**Alcohol use**					
Never or non-active drinker	212 (72.4)	99 (73.3)	113 (71.5)	3 (1.0)	0.79
Active drinker	81 (27.6)	36 (26.7)	45 (28.5)		
**Exercise frequency** (days/week) (*n* = 272)	1.6 ±1.6	1.5 ±1.5	1.7 ±1.6	3 (1.0)	0.41
**Marital status**					
Single	271 (92.5)	123 (91.1)	148 (93.7)	2 (0.6)	0.51
Married	22 (7.5)	12 (8.9)	10 (6.3)		
**Salary (THB/month)** (*n* = 275)	36830.5 ±13631.1	36339.1 ±13205.5	37258.5 ± 14022.0	21 (7.1)	0.58
**Financial status**					
Just enough	130 (44.4)	50 (37.0)	80 (50.6)	3 (1.0)	**0.018**
More than adequate	124 (42.3)	69 (51.1)	55 (34.8)		
Inadequate	39 (13.3)	16 (11.9)	23 (14.6)		
**Residency year**					
First-year	80 (27.2)	37 (27.0)	43 (27.4)	2 (0.7)	0.97
Second-year	94 (32.0)	43 (31.4)	51 (32.5)		
Third-year	78 (26.5)	36 (26.3)	42 (26.8)		
Fourth-year and more	42 (14.3)	21 (15.3)	21 (13.4)		
**Original affiliation**					
Ministry of Public Health	160 (54.1)	66 (48.2)	94 (59.1)	0 (0.0)	0.063
Ministry of Tertiary Education	136 (45.9)	71 (51.8)	65 (40.9)		
**Hospital departments**					
Other departments	126 (42.6)	60 (43.8)	66 (41.5)	0 (0.0)	0.72
Major departments	170 (57.4)	77 (56.2)	93 (58.5)		
**Total work hours** (hours/week) (*n* = 283)	75.0 ±21.8	74.9 ±19.9	75.0 ±23.4		0.96
**Psychiatric underlying disease**					
Not active	275 (93.2)	122 (89.7)	153 (96.2)	1 (0.3)	**0.035**
Active	20 (6.8)	14 (10.3)	6 (3.8)		
**Suicidal ideation in the past 12 months**					
No	278 (95.2)	122 (91.7)	156 (98.1)	4 (1.4)	**0.013**
Yes	14 (4.8)	11 (8.3)	3 (1.9)		
**Sleep duration (hours/day during last week)** (*n* = 278)	6.0 ±1.2	6.0 ±1.2	6.1 ±1.1	18 (6.1)	0.46
**Depressive symptoms**					
Mild	202 (69.4)	68 (51.1)	134 (84.8)	5 (1.7)	**<0.001**
Moderate to high	89 (30.6)	65 (48.9)	24 (15.2)		
**Severe medical error during the last 3 months**					
No	274 (95.1)	122 (92.4)	152 (97.4)	8 (2.7)	0.057
Yes	14 (4.9)	10 (7.6)	4 (2.6)		
**Non-severe medical error during the last 3 months**					
No	255 (88.5)	111 (84.1)	144 (92.3)	8 (2.7)	**0.040**
Yes	33 (11.5)	21 (15.9)	12 (7.7)		
**Medication prescription medical error during the last 3 months**					
No	231 (80.2)	97 (73.5)	134 (85.9)	8 (2.7)	**0.011**
Yes	57 (19.8)	35 (26.5)	22 (14.1)		
**Laboratory order medical error during the last 3 months**					
No	252 (87.5)	110 (83.3)	142 (91.0)	8 (2.7)	0.073
Yes	36 (12.5)	22 (16.7)	14 (9.0)		

SD, Standard deviation; THB, Thai Baht

Physicians reported an average daily work duration of 75.0 hours per week (SD ±21.8), with an average of 6.0 hours of sleep (SD ±1.2) in the past month. About 6.8% of physicians had active underlying psychiatric diseases, while 30.6% experienced moderate to high levels of depression, and 29.7% had underlying medical conditions. Fourteen participants (4.8%) reported suicidal ideation in the past 12 months. Furthermore, 4.9% and 11.5% of physicians acknowledged severe and non-severe medical errors in the past 3 months, while 19.8% and 12.5% reported medication prescription and laboratory errors. All types of errors demonstrated significant differences or trends between those with and without burnout (*p* = 0.05 to 0.073).

### Burnout syndrome

**Fig 2:**
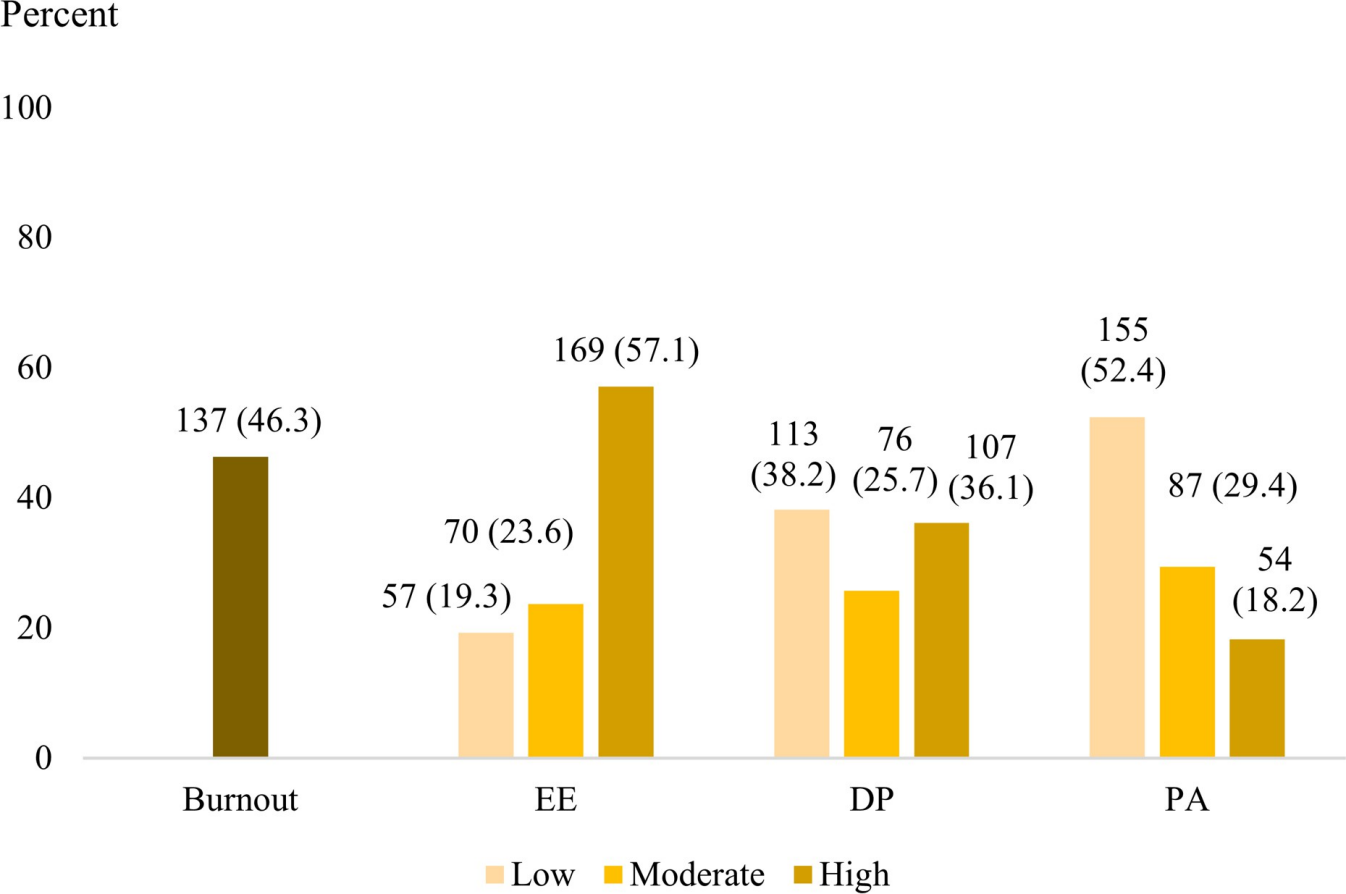
Prevalence of burnout and three subdomains among resident physicians; EE, Emotional exhaustion; DP, Depersonalized; PA, Personal accomplishment.

Nearly half of the participants (46.3%) reported experiencing burnout syndrome. When examining each domain, the prevalence varied across EE, DP, and low PA as follows: more than half were categorized as having high EE (57.1%), while approximately one-third experienced DP (36.1%), and roughly half reported low PA (52.4%) (Fig 2).

### Factors associated with burnout syndrome in medical residents

The factors associated with burnout, derived from four separate models using the confounder summary scores model, along with multiple imputations of missing data, are detailed in Table 2. For burnout and Table 3 for subdomains of burnout. Comparison of the complete case analysis was provided in S1 and S2 Tables. The result from the complete cases analysis demonstrated the similar association pattern of determinants, albeit with wider confidence interval due to the loss of power from the missing data, resulting in non-significant finding in the weakest associations, especially the medical error and low PA.

**Table 2:** Factors associated with burnout among resident physicians (multiple imputation, n = 296).

Determinants	Burnout		
	aOR (95%CI)	t-statistic	*p*-Value
**Model 1: Demographic determinants (F = 2.84, R**^**2**^ **= 0.074)**			
Gender			
Female	Reference		
Male	2.18 (1.30, 3.67)	2.93	**0.003**
Age, per 1-year increase	0.93 (0.84, 1.02)	-1.52	0.128
Marital status			
Single	Reference		
Married	1.95 (0.77, 4.91)	1.41	0.158
Have physical underlying disease	1.02 (0.59, 1.74)	0.06	0.950
Active smoker	13.23 (1.48, 118.00)	2.31	**0.021**
Active drinker	0.57 (0.33, 1.00)	-1.97	**0.049**
Exercise frequency, per 1-day/week increase	0.86 (0.72, 1.02)	-1.79	0.074
**Model 2: Working conditions (F = 1.44, R**^**2**^ **= 0.037)**			
Financial status			
Just enough	Reference		
More than adequate	0.48 (0.29, 0.81)	-2.75	**0.006**
Inadequate	0.56 (0.26, 1.21)	-1.48	0.140
Residency year			
First-year	Reference		
Second-year	1.01 (0.54, 1.87)	0.02	0.987
Third-year	0.98 (0.51, 1.88)	-0.06	0.956
Fourth-year and more	1.51 (0.69, 3.29)	1.04	0.297
Original affiliation			
Ministry of Public Health	Reference		
Ministry of Tertiary Education	0.62 (0.38, 1.00)	-1.96	**0.049**
Departments			
Other departments	Reference		
Major departments	0.82 (0.50, 1.33)	-0.81	0.415
Total work hours, per 1-hour increase	1.17 (0.69, 1.99)	0.59	0.552
Salary, per 10,000 THB increase	1.00 (0.82, 1.21)	0.01	0.992
**Model 3: Psychiatric determinants (F = 9.65, R**^**2**^ **= 0.127)**			
Active psychiatric underlying disease	1.80 (0.64, 5.03)	1.11	0.265
Had suicidal ideation in the last 12 months	2.95 (0.71, 12.34)	1.48	0.138
Sleep duration (hours/day during last week), per 1-hour increase	0.96 (0.77, 1.20)	-0.38	0.705
Depressive symptoms, moderate to high	4.94 (2.81, 8.69)	5.55	**<0.001**
**Model 4: Medical errors, during the last 3 months (F = 3.37, R**^**2**^ **= 0.013)**			
Severe errors	1.62 (0.68, 3.87)	1.08	0.281
Non-severe error	1.70 (0.45, 6.51)	0.78	0.435
Medication prescription error	1.87 (0.93, 3.76)	1.77	0.077
Laboratory order error	1.14 (0.49, 2.62)	0.30	0.765
**Confounder summary score; F = 61.13, R**^**2**^ **= 0.201, Hosmer-Lemeshow test for goodness-of-fit test (p = 0.587)**			
Demographic determinants	2.80 (1.68, 4.64)	3.98	**<0.001**
Working conditions	2.97 (1.54, 5.71)	3.26	**0.001**
Psychiatric determinants	2.47 (1.77, 3.45)	5.29	**<0.001**
Medical errors	2.14 (1.05, 4.34)	2.10	**0.035**

aOR, Adjusted odds ratio.

**Table 3:** Factors associated with each domain of burnout among resident physicians (multiple imputation, n = 296).

Determinants	High EE			High DP			Low PA		
	aOR (95%CI)	t-statistic	*p*-Value	aOR (95%CI)	t-statistic	*p*-Value	aOR (95%CI)	t-statistic	*p*-Value
**Model 1: Demographic determinants**	**F = 2.82, R**^**2**^ **= 0.066**			**F = 2.06, R**^**2**^ **= 0.042**			**F = 1.34, R**^**2**^ **= 0.034**		
**Gender**									
Female	Reference			Reference			Reference		
Male	1.05 (0.62, 1.77)	0.18	0.855	2.08 (1.23, 3.54)	2.72	**0.007**	1.39 (0.83, 2.33)	1.26	0.206
**Age**, per 1-year increase	0.97 (0.88, 1.07)	-0.56	0.577	0.95 (0.85, 1.06)	-0.94	0.347	1.03 (0.94, 1.13)	0.61	0.539
**Marital status**									
Single	Reference			Reference			Reference		
Married	2.75 (0.99, 7.60)	1.95	0.052	1.06 (0.40, 2.83)	0.11	0.909	0.54 (0.20, 1.45)	-1.23	0.220
**Have physical underlying disease**	1.98 (1.13, 3.48)	2.37	**0.018**	1.01 (0.58, 1.76)	0.04	0.967	0.91 (0.53, 1.55)	-0.35	0.728
**Active smoker**	5.41 (0.83, 35.26)	1.76	0.078	2.95 (0.71, 12.29)	1.49	0.137	11.75 (1.42, 96.95)	2.29	**0.022**
**Active drinker**	0.66 (0.38, 1.17)	-1.42	0.154	1.21 (0.69, 2.11)	0.67	0.505	0.77 (0.44, 1.34)	-0.93	0.351
**Exercise frequency**, per 1-day/week increase	0.81 (0.68, 0.96)	-2.38	**0.017**	0.94 (0.80, 1.12)	-0.65	0.513	0.91 (0.77, 1.08)	-1.10	0.272
**Model 2: Working conditions**	**F = 1.67, R**^**2**^ **= 0.037**			**F = 1.17, R**^**2**^ **= 0.026**			**F = 0.90, R**^**2**^ **= 0.029**		
**Financial status**									
Just enough	Reference			Reference			Reference		
More than adequate	0.48 (0.28, 0.81)	-2.73	**0.006**	0.65 (0.38, 1.10)	-1.61	0.108	0.69 (0.41, 1.16)	-1.40	0.162
Inadequate	0.53 (0.25, 1.13)	-1.65	0.099	0.64 (0.29, 1.43)	-1.08	0.280	0.83 (0.40, 1.74)	-0.48	0.629
**Residency year**									
First-year	Reference			Reference			Reference		
Second-year	1.79 (0.94, 3.41)	1.76	0.078	0.93 (0.49, 1.79)	-0.20	0.839	0.97 (0.52, 1.79)	-0.11	0.914
Third-year	0.79 (0.42, 1.52)	-0.70	0.485	1.36 (0.70, 2.65)	0.90	0.369	0.87 (0.46, 1.66)	-0.41	0.682
Fourth-year	1.66 (0.75, 3.69)	1.25	0.212	2.13 (0.96, 4.72)	1.87	0.062	1.08 (0.49, 2.37)	0.19	0.853
**Original affiliation**									
Ministry of Public Health	Reference			Reference			Reference		
Ministry of Tertiary Education	0.83 (0.51, 1.36)	-0.73	0.467	0.68 (0.41, 1.12)	-1.51	0.132	0.57 (0.36, 0.92)	-2.28	**0.022**
**Departments**									
Other departments	Reference			Reference			Reference		
Major departments	0.81 (0.49, 1.34)	-0.81	0.420	0.69 (0.42, 1.15)	-1.42	0.155	0.83 (0.52, 1.35)	-0.74	0.462
**Total work hours,** per 1-hour increase	1.16 (0.67, 2.03)	0.52	0.600	1.32 (0.77, 2.26)	1.00	0.317	0.87 (0.51, 1.49)	-0.50	0.616
**Salary,** per 10,000 THB increase	0.92 (0.77, 1.11)	-0.83	0.407	1.08 (0.89, 1.31)	0.75	0.453	1.01 (0.85, 1.22)	0.16	0.876
**Model 3: Psychiatric determinants**	**F = 10.42, R**^**2**^ **= 0.165**			**F = 5.98, R**^**2**^ **= 0.074**			**F = 3.26, R**^**2**^ **= 0.036**		
**Active psychiatric underlying disease**	1.69 (0.46, 6.23)	0.79	0.430	1.47 (0.53, 4.11)	0.74	0.461	1.27 (0.49, 3.31)	0.49	0.621
**Had suicidal ideation in the last 12 months**	6.64 (0.87, 50.69)	1.82	0.068	3.54 (0.98, 12.77)	1.93	0.054	1.31 (0.44, 3.90)	0.48	0.630
**Sleep duration** (hours/day during last week), per 1 hour increased	0.94 (0.74, 1.19)	-0.52	0.604	0.94 (0.75, 1.19)	-0.48	0.629	1.09 (0.89, 1.34)	0.85	0.398
**Depressive symptoms**, moderate to high	7.58 (3.82, 15.01)	5.80	**<0.001**	3.08 (1.80, 5.27)	4.12	**<0.001**	2.38 (1.40, 4.07)	3.18	**0.001**
**Model 4: Medical error, compared to having no medical error during the last 3 months**	**F = 3.62, R**^**2**^ **= 0.042**			**F = 5.30, R**^**2**^ **= 0.074**			**F = 0.46, R**^**2**^ **= 0.004**		
**Severe medical error**	2.59 (0.87, 7.65)	1.72	0.086	1.92 (0.84, 4.40)	1.55	0.121	0.73 (0.30, 1.79)	-0.69	0.488
**Non-severe medical error**	1.11 (0.22, 5.50)	0.13	0.900	1.60 (0.45, 5.78)	0.72	0.470	2.21 (0.57, 8.61)	1.14	0.253
**Medication prescription medical error**	2.75 (1.26, 5.98)	2.54	**0.011**	2.92 (1.46, 5.82)	3.04	**0.002**	0.98 (0.48, 1.99)	-0.05	0.959
**Laboratory order medical error**	0.77 (0.31, 1.90)	-0.57	0.569	0.93 (0.41, 2.07)	-0.19	0.851	1.15 (0.48, 2.75)	0.32	0.749
**Confounder summary score**	**F = 54.31, R**^**2**^ **= 0.250, Hosmer-Lemeshow test for goodness-of-fit test (p = 0.052)**			**F = 46.74, R**^**2**^ **= 0.173, Hosmer-Lemeshow test for goodness-of-fit test (p = 0.433)**			**F = 28.34, R**^**2**^ **= 0.080, Hosmer-Lemeshow test for goodness-of-fit test (p = 0.489)**		
Demographic determinants	2.78 (1.58, 4.89)	3.54	**<0.001**	2.63 (1.44, 4.80)	3.14	**0.002**	2.65 (1.30, 5.40)	2.69	**0.007**
Working conditions	2.95 (1.54, 5.66)	3.25	**0.001**	3.11 (1.57, 6.14)	3.27	**0.001**	2.51 (1.22, 5.17)	2.50	**0.012**
Psychiatric determinants	2.57 (1.88, 3.52)	5.87	**<0.001**	2.42 (1.57, 3.74)	3.98	**<0.001**	2.45 (1.39, 4.32)	3.10	**0.002**
Medical errors	2.88 (1.56, 5.32)	3.37	**0.001**	2.57 (1.52, 4.34)	3.53	**<0.001**	1.64 (0.28, 9.75)	0.54	0.588

aOR, Adjusted odds ratio.

### The first domain: Demographic determinants

Demographic determinants were associated with burnout (aOR 2.80 95% CI 1.68–4.64), high EE (aOR 2.78, 95% CI 1.58–4.89), high DP (aOR 2.63, 95% CI 1.44–4.80), and low PA (aOR 2.65, 95% CI 1.30–5.40). Specifically, higher odds of burnout were associated with being an active smoker (adjusted odds Ratio (aOR) 13.23, 95% CI 1.48–118.00), male gender (aOR 2.18, 95% CI 1.30–3.67), and having a physical underlying disease (aOR 1.98, 95% CI 1.13–3.48). Whereas high DP was associated with male gender (aOR 2.08, 95% CI 1.23–3.54). In terms of PA, low levels were associated with smoking (aOR 11.75, 95% CI 1.42–96.95)

### The second domain: Working conditions

Working conditions were significantly associated with burnout (aOR 2.97, 95% CI 1.54–5.71), high EE (aOR 2.95, 95% CI 1.54–5.66), high DP (aOR 3.11, 95% CI 1.57–6.14), and low PA (aOR 2.51, 95% CI 1.22–5.17). In details, having more than adequate financial status (aOR 0.48, 95% CI 0.29–0.81), being an active drinker (aOR 0.57, 95% CI 0.33–1.00), and working in the Ministry of Tertiary Education (aOR 0.62, 95% CI 0.38–1.00) demonstrated lower odds of burnout. Moreover, lower odds of burnout in more than adequate income subgroup were observed (aOR 0.48, 95% CI 0.28–0.81). Whereas original affiliation from a university hospital was associated with lower odds for low PA (aOR 0.57, 95% CI 0.36–0.92).

### The third domain: Psychiatric determinants

Psychiatric determinants were associated with burnout (aOR 2.47, 95% CI 1.77–3.45), high EE (aOR 2.57, 95% CI 1.88–3.52), high DP (aOR 2.42, 95% CI 1.57–3.74), and low PA (aOR 2.45, 95% CI 1.39–4.32). Components of psychiatric determinants consisting of moderate to high depressive symptoms were positively associated with burnout (aOR 4.94, 95% CI 2.81–8.69), high EE (aOR 7.58, 95% CI 3.82–15.01), high DP (aOR 3.08, 95% CI 1.80–5.27), and low PA (aOR 2.38, 95% CI 1.40–4.07).

### The fourth domain: Medical errors

Medical errors were significantly associated with burnout (aOR 2.14, 95% CI 1.05–4.34), high EE (aOR 2.88, 95%CI 1.56–5.32), and high DP (aOR 2.57, 95%CI 1.52–4.34). Regarding the characteristics of medical error, medical prescription errors were associated with high EE (aOR 2.75, 95% CI 1.26–5.98) and high DP (aOR 2.92, 95% CI 1.46–5.82).

### The confounder summary score models

In summary, all four determinant domains were positively associated with burnout syndrome. In each subdomain of burnout, all four determinant subdomains exhibited a positive association with high EE and high DP. Nevertheless, only three out of four determinant domains, except medical errors, were positively associated with low PA.

## Discussion

In this study, 46.3% of resident physicians experienced burnout, with 57.1% reporting high levels of EE, 36.1% exhibiting high levels of DP, and 52.4% showing low levels of PA. Our results indicated a higher prevalence of burnout in our study than most studies conducted in various training center across Thailand (0% to 10.7%) [9, 11, 33]. When considering into each subdomains, high EE (17.0% to 53.0%) [9–12, 15, 33] and high DP (12.0–45.5%) [9–12, 15, 33] was also higher than all studies, albeit a similar prevalence of low PA with previous study was found (0% to 32.8%) [9–11, 15, 33]. Internationally, burnout prevalence in our study were higher than other studies (11.3% to 41.0%) [34–38], except for one study involving Irish urologists in training (52.0%) [39], which did not use low PA as a criterion for burnout syndrome. Subdomains including high EE (28.6% to 32.5%) [38, 39] and high DP also exhibit considerably higher prevalence (20.0% to 26.9%) [38, 39]. However, low PA prevalence was found to be lower (32.8% to 40.3%) [38, 39]. Resultantly, both in Thailand and internationally, inconsistencies in reporting overall burnout syndrome across studies [10, 12, 15, 34–36, 39] made comparisons challenging, especially compared to the relatively complete reporting of each domain, especially EE, DP, and PA. The higher prevalence observed in our study may be attributed to factors specific to our training center, including its learning and working conditions, as well as differences in the proportion and composition of medical specialties compared to other training centers, accompanying with the data collection time frame that encompass COVID-19 pandemic [40, 41]. Moreover, the disparity in findings may be attributed to our distinctive modeling approach. Unlike other previous studies that examined each factor individually using single binary logistic regression model, our study employed a confounder summary score, allowing for simultaneous comparison of determinants across all three domains.

Demographic determinants demonstrated strong association with burnout, high EE, high DP, and low PA. Regarding the components of demographic determinants, A strong association was found between active smoking status and burnout syndrome and low PA, which was consistent with findings from global studies [3, 4, 7]. The role of smoking in contributing to overall burnout and low PA can be explained by the theory of self-medication. According to this theory, burnout, which shares similarities with depression, may lead individuals to use nicotine-based substances to alleviate negative symptoms, particularly by improving concentration, activeness, and alertness [42, 43]. Additionally, another unhealthy behavior–low exercise frequency–was associated with both overall burnout and high EE. Interesting, our study found a negative association between female gender and both overall burnout and DP, which contrasts with some previous studies that either suggested a potentially associated with burnout [44] or found no associated [45]. This unique approach highlights the distinct and significant role that female gender plays among demographic determinants [44, 45].

Working conditions were associated with burnout, high EE, high DP, and low PA. Some of its components revealed a significant association with burnout especially for income and burnout, particularly in terms of relative adequacy and savings, while the absolute income amount did not show a significant association. This result may be partially explained by the uniform pay structure for resident physicians, with minimal differences across academic years, except for those taking on additional work in private hospitals. However, such private work is often limited by the already high workload within the training program. The policy implication here is twofold: first, ensuring the well-being and retention of the health workforce is crucial for maintaining a sustainable healthcare system; second, offering competitive compensation packages is vital for attracting and retaining physicians [43]. Regarding affiliation, our hypothesis considered that the working environment [46] and the experience level [47] of physicians from different specialties contribute to personal accomplishment. In the Thai resident training system, there are two distinct career pathways funded by the MHESI and MOPH. The first pathway recruits newly graduated general practitioners for training in university hospitals immediately after a one-year internship. The second is a grant-based training program in government hospitals designed for specialists, allowing general practitioners to train in rural government hospitals for three years before becoming eligible for residency training [42, 43]. This dual-track residency training suggests that having more clinical experience may act as a protective factor in dealing with academic and psychological challenges during the training process [46]. Consequently, strong social support during the early career stages becomes crucial [47]. A supportive environment with colleagues and leaders facilitating discussions and providing an outlet for resident physician concerns [48] could potentially mitigate the burnout [49] by balancing psychological burdens and promoting resilience [50].

In our study, psychiatric determinants were associated with burnout, high EE, high DP, and low PA. Depressive symptoms in particular emerged as a major contributing factor associated with burnout syndrome, high EE, high DP, and low PA. The well-established association between psychiatric well-being and burnout syndrome, observed in both the general population and healthcare personnel [5], underscores the importance of addressing mental health in the context of burnout. Additionally, other conditions similar to burnout may modify this relationship. We suspected that the co-occurrence of chronic stress with burnout could act as a modifier, potentially precipitating clinical depression.

Initially, medical errors were hypothesized to be strongly related to overall burnout in this study, aligning with findings in other occupational hazards studies [49, 51, 52]. However, the association was found to be weakly associated with burnout and its subdomains of high EE and high DP, albeit no association was found with low PA. The association was also limited to the particular type of medical error concerning the medication prescription errors, which impact the odds of having high EE and high DP, but also not associated with low PA. When compared to four other domains, medical errors were the domain with smallest association with the burnout syndrome, which probably resulted from larger explanation of burnout syndrome from the third domain, psychiatric determinants. this finding of attenuated association of medical errors aligns with a large study in the US involving the broader physician population, supporting the evidence that overall burnout may not directly related to medical errors but may be linked to underlying issues such as depression [5].

### Strengths and limitations

Our study has several limitations. Firstly, cross-sectional design introduces temporal ambiguity, making it difficult to establish casualty. Secondly, our ability to discern the effect of each individual variable is limited. The confounder summary score method relies on predicted values from a set of variables in four separate models, allowing comparison only in terms of the overall effect of a group of variables within each domain rather than individual variables. Despite these limitations, the study’s strength lies in the use of a comprehensive, multi-dimensional, standardized questionnaire for psychological assessment. Along with a high response rate. Moreover, multiple imputations were carried out and ultimately able to provide generally similar logit coefficients and narrower confidence intervals compared to the complete case analyses. By including all specialties and academic years in residency training programs across all institutes, our findings can be applied broadly, including to other hospitals with similar characteristics.

## Conclusions

This study reveals that nearly half of all medical residents in a university hospital in Thailand experienced high levels of burnout syndrome. All four domains: demographic determinants, working conditions, psychiatric determinants, and medical errors were associated with burnout, high EE, and high DP. Except for medical errors, the other three domains were also positively associated with low PA. Thereby, medical residency training programs should establish a supportive system that actively monitors and addresses depressive symptoms while fostering an environment conductive to academic discussions. Implementing preventive measures, such as increasing pay rates, employee assistant program and risk management to prevent medical errors, such as implementation of electronic medical records, could play a crucial role in mitigating burnout among resident physicians. Further research is needed to explore the most effective strategies for promoting the well-being of medical residents and preventing burnout.

## Supporting information

S1 Table Factors associated with burnout among resident physicians (complete case analysis). (DOCX)

S2 Table Factors associated with each domain of burnout among resident physicians (complete case analysis). (DOCX)

## List of abbreviations

aOR: Adjusted odds ratio
CI: Confidence interval
DP: Depersonalization
EE: Emotional exhaustion
MHESI: Ministry of Higher Education, Science, Research and Innovation
MOPH: Ministry of Public Health
PA: Personal accomplishment
SD: Standard deviation

## References

[1] Maslach C, Leiter MP. Understanding the burnout experience: recent research and its implications for psychiatry. 2016;15(2):103–11. doi: 10.1002/wps.20311 27265691 PMC4911781

[2] Maslach C, Jackson S, Leiter M. The Maslach Burnout Inventory Manual. 31997. p. 191–218.

[3] Alqahtani AM, Awadalla NJ, Alsaleem SA, Alsamghan AS, Alsaleem MA. Burnout Syndrome among Emergency Physicians and Nurses in Abha and Khamis Mushait Cities, Aseer Region, Southwestern Saudi Arabia. TheScientificWorldJournal. 2019;2019:4515972. doi: 10.1155/2019/4515972 30906233 PMC6398028

[4] Xia L, Jiang F, Rakofsky J, Zhang Y, Zhang K, Liu T, et al. Cigarette Smoking, Health-Related Behaviors, and Burnout Among Mental Health Professionals in China: A Nationwide Survey. Frontiers in psychiatry. 2020;11:706. doi: 10.3389/fpsyt.2020.00706 32765329 PMC7379885

[5] Menon NK, Shanafelt TD, Sinsky CA, Linzer M, Carlasare L, Brady KJS, et al. Association of Physician Burnout With Suicidal Ideation and Medical Errors. JAMA Network Open. 2020;3(12):e2028780-e. doi: 10.1001/jamanetworkopen.2020.28780 33295977 PMC7726631

[6] Brown SD, Goske MJ, Johnson CM. Beyond substance abuse: stress, burnout, and depression as causes of physician impairment and disruptive behavior. J Am Coll Radiol. 2009;6(7):479–85. doi: 10.1016/j.jacr.2008.11.029 19560063

[7] Petrelli F, Scuri S, Tanzi E, Nguyen C, Grappasonni I. Public health and burnout: a survey on lifestyle changes among workers in the healthcare sector. Acta bio-medica: Atenei Parmensis. 2018;90(1):24–30. doi: 10.23750/abm.v90i1.7626 30889151 PMC6502147

[8] Choon-ngarm N. Mental health and burnout among physicians in general hospital and community hospital in Nakhon Ratchasima province. Journal of Mental Health of Thailand. 2020;28(4):348–59.

[9] Srikam SJ, V; Lalitananantphong D. Job Burnout and Related Factors among Residents of King Chulalongkorn Memorial Hospital. J Psychiatr Assoc Thailand. 2014;59(2):139–50.

[10] Thamrongvisava SP C. The Prevalence and Associated Factors of Burnout Syndrome among Residents in Training at Faculty of Medicine, Songklanagarind Hospital. J Psychiatr Assoc Thailand 2018. 2018;63(4):309–20.

[11] Charoentanyarak A, Anothaisintawee T, Kanhasing R, Poonpetcharat P. Prevalence of Burnout and Associated Factors Among Family Medicine Residency in Thailand. Journal of medical education and curricular development. 2020;7:2382120520944920. doi: 10.1177/2382120520944920 32782930 PMC7388096

[12] Nimmawitt N, Wannarit K, Pariwatcharakul P. Thai psychiatrists and burnout: A national survey. PloS one. 2020;15(4):e0230204. doi: 10.1371/journal.pone.0230204 32315309 PMC7173626

[13] Tuangpermsub R, Nuallaong W, Charernboon T. Burnout and Related Factor among Residents of Thammasat University Hospital In the COVID-19 outbreak. Journal of the Psychiatric Association of Thailand. 2021;66(2):189–202.

[14] Yothaburi SA N; Yurayat P;. Prevalence and Associated Factors of Burnout Syndrome in Mahasarakham Hospital Employee. Mahasarakham Hospital Journal. 2022;19(3):149–64.

[15] Na Bangxang J. Resident Burnout: Prevalence and Associated Factors in Rajavithi Hospital. Journal of the Psychiatric Association of Thailand. 2019;64(1):61–76.

[16] Accreditation Council for Graduate Medical Education. Common Program Requirements (Residency). 2023.

[17] The Medical Council of Thailand. List of Post Graduate Recognized Institutions 2024 [Available from: https://tmc.or.th/En/post_graduate.php].

[18] Low ZX, Yeo KA, Sharma VK, Leung GK, McIntyre RS, Guerrero A, et al. Prevalence of Burnout in Medical and Surgical Residents: A Meta-Analysis. International journal of environmental research and public health. 2019;16(9). doi: 10.3390/ijerph16091479 31027333 PMC6539366

[19] Mangory KY, Ali LY, Rø KI, Tyssen R. Effect of burnout among physicians on observed adverse patient outcomes: a literature review. BMC health services research. 2021;21(1):369. doi: 10.1186/s12913-021-06371-x 33879135 PMC8057942

[20] Harris PA, Taylor R, Minor BL, Elliott V, Fernandez M, O’Neal L, et al. The REDCap consortium: Building an international community of software platform partners. Journal of Biomedical Informatics. 2019;95:103208. doi: 10.1016/j.jbi.2019.103208 31078660 PMC7254481

[21] Harris PA, Taylor R, Thielke R, Payne J, Gonzalez N, Conde JG. Research electronic data capture (REDCap)—A metadata-driven methodology and workflow process for providing translational research informatics support. Journal of Biomedical Informatics. 2009;42(2):377–81. doi: 10.1016/j.jbi.2008.08.010 18929686 PMC2700030

[22] Surawattanasakul V, Kiratipaisarl W, Siviroj P. Burnout and Quality of Work Life among Physicians during Internships in Public Hospitals in Thailand. 2024;14(5):361. doi: 10.3390/bs14050361 38785852 PMC11117651

[23] Sammawart S. Burnout among nurses in Ramathibodi Hospital. Bangkok, Thailand: Mahidol University; 1989.

[24] Meehan PJ, Lamb JA, Saltzman LE, O’Carroll PW. Attempted suicide among young adults: progress toward a meaningful estimate of prevalence. The American journal of psychiatry. 1992;149(1):41–4. doi: 10.1176/ajp.149.1.41 1728183

[25] Panyawong W, Pavasuthipaisit C, Santitadakul R. Validation of the Thai Version of the Patient Health Questionnaire for Adolescents (PHQ-A) in adolescent psychiatric patients: Validation of the Thai version of the PHQ-A. International Journal of Child Development and Mental Health. 2020;8(1):30–40.

[26] Trockel M, Bohman B, Lesure E, Hamidi MS, Welle D, Roberts L, et al. A Brief Instrument to Assess Both Burnout and Professional Fulfillment in Physicians: Reliability and Validity, Including Correlation with Self-Reported Medical Errors, in a Sample of Resident and Practicing Physicians. Academic psychiatry: the journal of the American Association of Directors of Psychiatric Residency Training and the Association for Academic Psychiatry. 2018;42(1):11–24. doi: 10.1007/s40596-017-0849-3 29196982 PMC5794850

[27] Vittinghoff E, McCulloch CE. Relaxing the Rule of Ten Events per Variable in Logistic and Cox Regression. American Journal of Epidemiology. 2007;165(6):710–8. doi: 10.1093/aje/kwk052 17182981

[28] van Smeden M, Moons KGM, de Groot JAH, Collins GS, Altman DG, Eijkemans MJC, et al. Sample size for binary logistic prediction models: Beyond events per variable criteria. Statistical Methods in Medical Research. 2018;28(8):2455–74. doi: 10.1177/0962280218784726 29966490 PMC6710621

[29] van Smeden M, de Groot JAH, Moons KGM, Collins GS, Altman DG, Eijkemans MJC, et al. No rationale for 1 variable per 10 events criterion for binary logistic regression analysis. BMC Medical Research Methodology. 2016;16(1):163. doi: 10.1186/s12874-016-0267-3 27881078 PMC5122171

[30] Greenland S. Confounder Summary Score. Wiley StatsRef: Statistics Reference Online. p. 1–3.

[31] Cadarette SM, Gagne JJ, Solomon DH, Katz JN, Stürmer T. Confounder summary scores when comparing the effects of multiple drug exposures. Pharmacoepidemiology and drug safety. 2010;19(1):2–9. doi: 10.1002/pds.1845 19757416 PMC2800174

[32] von Elm E, Altman DG, Egger M, Pocock SJ, Gøtzsche PC, Vandenbroucke JP. The Strengthening the Reporting of Observational Studies in Epidemiology (STROBE) statement: guidelines for reporting observational studies. Annals of internal medicine. 2007;147(8):573–7. doi: 10.7326/0003-4819-147-8-200710160-00010 17938396

[33] Puranitee P, Stevens FFCJ, Pakakasama S, Plitponkarnpim A, Vallibhakara SA-O, Busari JO, et al. Exploring burnout and the association with the educational climate in pediatric residents in Thailand. BMC Medical Education. 2019;19(1):245. doi: 10.1186/s12909-019-1687-7 31277615 PMC6612205

[34] Nishimura K, Nakamura F, Takegami M, Fukuhara S, Nakagawara J, Ogasawara K, et al. Cross-sectional survey of workload and burnout among Japanese physicians working in stroke care: the nationwide survey of acute stroke care capacity for proper designation of comprehensive stroke center in Japan (J-ASPECT) study. Circulation Cardiovascular quality and outcomes. 2014;7(3):414–22. doi: 10.1161/CIRCOUTCOMES.113.000159 24823957

[35] Twellaar M, Winants Y, Houkes I. How healthy are Dutch general practitioners? Self-reported (mental) health among Dutch general practitioners. The European journal of general practice. 2008;14(1):4–9. doi: 10.1080/13814780701814911 18464166

[36] van der Wal RA, Bucx MJ, Hendriks JC, Scheffer GJ, Prins JB. Psychological distress, burnout and personality traits in Dutch anaesthesiologists: A survey. European journal of anaesthesiology. 2016;33(3):179–86. doi: 10.1097/EJA.0000000000000375 26575009

[37] Upton D, Mason V, Doran B, Solowiej K, Shiralkar U, Shiralkar S. The experience of burnout across different surgical specialties in the United Kingdom: a cross-sectional survey. Surgery. 2012;151(4):493–501. doi: 10.1016/j.surg.2011.09.035 22088818

[38] Saijo Y, Chiba S, Yoshioka E, Kawanishi Y, Nakagi Y, Itoh T, et al. Effects of work burden, job strain and support on depressive symptoms and burnout among Japanese physicians. International journal of occupational medicine and environmental health. 2014;27(6):980–92. doi: 10.2478/s13382-014-0324-2 25503892

[39] O’Kelly F, Manecksha RP, Quinlan DM, Reid A, Joyce A, O’Flynn K, et al. Rates of self-reported ’burnout’ and causative factors amongst urologists in Ireland and the UK: a comparative cross-sectional study. BJU international. 2016;117(2):363–72. doi: 10.1111/bju.13218 26178315

[40] Daryanto B, Rahmadiani N, Amorga R, Kautsarani I, Susilo H, Persada Isma SP. Burnout syndrome among residents of different surgical specialties in a tertiary referral teaching hospital in Indonesia during COVID-19 pandemic. Clinical Epidemiology and Global Health. 2022;14:100994. doi: 10.1016/j.cegh.2022.100994 35155847 PMC8824714

[41] Balch CM, Shanafelt TD, Sloan JA, Satele DV, Freischlag JA. Distress and career satisfaction among 14 surgical specialties, comparing academic and private practice settings. Ann Surg. 2011;254(4):558–68. doi: 10.1097/SLA.0b013e318230097e 21946217

[42] Intralawan D, Morikawa HC, Morikawa MJ, Porruan R. Focusing on the assets in our challenges: family medicine residency programme in Chiang Rai, Thailand. Family medicine and community health. 2020;8(4). doi: 10.1136/fmch-2020-000500 33060127 PMC7566419

[43] Prueksaritanond S, Tuchinda P. General practice residency training program in Thailand: past, present, and future. Journal of the Medical Association of Thailand = Chotmaihet thangphaet. 2001;84(8):1153–7. 11758852

[44] Alrawashdeh HM, Al-Tammemi AB, Alzawahreh MK, Al-Tamimi A, Elkholy M, Al Sarireh F, et al. Occupational burnout and job satisfaction among physicians in times of COVID-19 crisis: a convergent parallel mixed-method study. BMC public health. 2021;21(1):811. doi: 10.1186/s12889-021-10897-4 33906619 PMC8079229

[45] Rao S, Ferris TG, Hidrue MK, Lehrhoff SR, Lenz S, Heffernan J, et al. Physician Burnout, Engagement and Career Satisfaction in a Large Academic Medical Practice. (1554–6179 (Electronic)). 10.3121/cmr.2019.1516 PMC7153796 31959669

[46] Hodkinson A, Zhou A, Johnson J, Geraghty K, Riley R, Zhou A, et al. Associations of physician burnout with career engagement and quality of patient care: systematic review and meta-analysis. BMJ (Clinical research ed). 2022;378:e070442. doi: 10.1136/bmj-2022-070442 36104064 PMC9472104

[47] del Carmen MG, Herman J, Rao S, Hidrue MK, Ting D, Lehrhoff SR, et al. Trends and Factors Associated With Physician Burnout at a Multispecialty Academic Faculty Practice Organization. JAMA Network Open. 2019;2(3):e190554-e. 30874776 10.1001/jamanetworkopen.2019.0554 PMC6484653

[48] Sargent MC, Sotile W, Sotile MO, Rubash H, Barrack RL. Stress and Coping Among Orthopaedic Surgery Residents and Faculty. 2004;86(7):1579–86. 10.2106/00004623-200407000-00032 15252111

[49] Yuan JH, Huang Y, Rosgen BK, Donnelly S, Lan X, Katz SJ. Burnout and fatigue amongst internal medicine residents: A cross-sectional study on the impact of alternative scheduling models on resident wellness. PloS one. 2023;18(9):e0291457. doi: 10.1371/journal.pone.0291457 37708198 PMC10501672

[50] Ozbay F, Johnson DC, Dimoulas E, Morgan CA, Charney D, Southwick S. Social support and resilience to stress: from neurobiology to clinical practice. Psychiatry (Edgmont (Pa: Township)). 2007;4(5):35–40. 20806028 PMC2921311

[51] Trockel MT, Menon NK, Rowe SG, Stewart MT, Smith R, Lu M, et al. Assessment of Physician Sleep and Wellness, Burnout, and Clinically Significant Medical Errors. JAMA Network Open. 2020;3(12):e2028111-e. doi: 10.1001/jamanetworkopen.2020.28111 33284339 PMC12064096

[52] Hall LH, Johnson J, Watt I, Tsipa A, O’Connor DB. Healthcare Staff Wellbeing, Burnout, and Patient Safety: A Systematic Review. PloS one. 2016;11(7):e0159015. doi: 10.1371/journal.pone.0159015 27391946 PMC4938539

