# Supplementary material for: Resident physician burnout and association with working conditions, psychiatric determinants, and medical errors: A cross-sectional study
Source: PLoS One. 2024 Oct 30;19(10):e0312839. doi: 10.1371/journal.pone.0312839 (PMC11524500; doi:10.1371/journal.pone.0312839)
Supplement: S1 Table — (DOCX) [file pone.0312839.s001.docx]

**S1 Table.** Factors associated with burnout among resident physicians (complete case analysis).

| **Determinants** | **Burnout** | |
| --- | --- | --- |
|  | **aOR (95%CI)** | ***p*-Value** |
| **Model 1: Demographic determinants (*n* = 271)** | | |
| **Gender** |  |  |
| Female | Reference |  |
| Male | 2.00 (0.29, 0.87) | **0.015** |
| **Age, per 1-year increase** | 0.91 (0.82, 1.01) | 0.084 |
| **Marital status** |  |  |
| Single | Reference |  |
| Married | 2.38 (0.87, 6.48) | 0.090 |
| **Have physical underlying disease** | 1.02 (0.58, 1.78) | 0.947 |
| **Active smoker** | 16.69 (1.92, 144.87) | **0.011** |
| **Active drinker** | 0.49 (0.26, 0.90) | **0.022** |
| **Exercise frequency,** per 1-day/week increase | 0.85 (0.72, 1.01) | 0.070 |
| **Model 2: Working conditions (*n* = 262)** | | |
| **Financial status** |  |  |
| Just enough | Reference |  |
| More than adequate | 0.43 (0.25, 0.74) | **0.003** |
| Inadequate | 0.59 (0.27, 1.30) | 0.191 |
| **Residency year** |  |  |
| First-year | Reference |  |
| Second-year | 0.98 (0.51, 1.89) | 0.958 |
| Third-year | 0.81 (0.41, 1.62) | 0.552 |
| Fourth-year | 1.19 (0.50, 2.79) | 0.695 |
| **Original affiliation** |  |  |
| Ministry of Public Health | Reference |  |
| Ministry of Tertiary Education | 1.50 (0.89, 2.52) | 0.128 |
| **Departments** |  |  |
| Other departments | Reference |  |
| Major departments | 0.86 (0.51, 1.45) | 0.575 |
| **Total work hours,** per 1-hour increase | 1.00 (0.99, 1.01) | 0.730 |
| **Salary, per 10,000 THB increase** | 1.01 (0.84, 1.22) | 0.922 |
| **Model 3: Psychiatric determinants (*n* = 274)** | | |
| **Active psychiatric underlying disease** | 1.93 (0.63, 5.90) | 0.252 |
| **Had suicidal ideation in the last 12 months** | 2.75 (0.65, 11.56) | 0.167 |
| **Sleep duration (hours/day during last week),**  per 1-hour increase | 0.93 (0.74, 1.18) | 0.564 |
| **Depressive symptoms, moderate to high** | 5.37 (3.00, 9.61) | **<0.001** |
| **Model 4: Medical errors, compared to having no medical error during the last 3 months (*n* = 288)** | | |
| Severe errors | 1.70 (0.40, 7.15) | 0.472 |
| Non-severe error | 1.58 (0.63, 3.95) | 0.327 |
| Medication prescription error | 1.86 (0.91, 3.80) | 0.087 |
| Laboratory order error | 1.14 (0.47, 2.75) | 0.766 |
| **Confounder summary score (*n* = 238)** | | |
| Demographic determinants | 2.44 (1.46, 4.07) | **0.001** |
| Working conditions | 2.64 (1.34, 5.22) | **0.005** |
| Psychiatric determinants | 2.41 (1.67, 3.47) | **<0.001** |
| Medical errors | 1.60 (0.72, 3.55) | 0.244 |

aOR, Adjusted odds ratio.
