# Supplementary material for: Resident physician burnout and association with working conditions, psychiatric determinants, and medical errors: A cross-sectional study
Source: PLoS One. 2024 Oct 30;19(10):e0312839. doi: 10.1371/journal.pone.0312839 (PMC11524500; doi:10.1371/journal.pone.0312839)
Supplement: S2 Table — (DOCX) [file pone.0312839.s002.docx]

**S2 Table.** Factors associated with each domain of burnout among resident physicians (complete case analysis).

| **Determinants** | **High EE** | | **High DP** | | **Low PA** | |
| --- | --- | --- | --- | --- | --- | --- |
|  | **aOR (95%CI)** | ***p*-Value** | **aOR (95%CI)** | ***p*-Value** | **aOR (95%CI)** | ***p*-Value** |
| **Model 1: Demographic determinants (n = 271)** | | | | | | |
| **Gender** |  |  |  |  |  |  |
| Male | Reference |  |  |  |  |  |
| Female | 0.99 (0.57, 1.70) | 0.967 | 0.54 (0.31, 0.94) | 0.029 | 0.76 (0.45, 1.29) | 0.303 |
| **Age**, per 1-year increase | 0.96 (0.86, 1.06) | 0.402 | 0.92 (0.83, 1.03) | 0.165 | 1.02 (0.93, 1.13) | 0.657 |
| **Marital status** |  |  |  |  |  |  |
| Single | Reference |  |  |  |  |  |
| Married | 2.71 (0.93, 7.92) | 0.068 | 1.29 (0.47, 3.52) | 0.617 | 0.63 (0.24, 1.66) | 0.345 |
| **Have physical underlying disease** | 1.86 (1.05, 3.29) | 0.033 | 1.04 (0.59, 1.84) | 0.887 | 0.96 (0.56, 1.66) | 0.889 |
| **Active smoker** | 5.80 (1.07, 31.28) | 0.041 | 3.56 (0.84, 15.03) | 0.084 | 12.27 (1.45, 103.72) | 0.021 |
| **Active drinker** | 0.69 (0.38, 1.24) | 0.209 | 1.12 (0.61, 2.03) | 0.719 | 0.72 (0.41, 1.28) | 0.263 |
| **Exercise frequency**, per 1-day/week increase | 0.80 (0.68, 0.95) | 0.013 | 0.94 (0.79, 1.12) | 0.495 | 0.91 (0.77, 1.08) | 0.270 |
| **Model 2: Working conditions (*n* = 262)** | | | | | | |
| **Financial status** |  |  |  |  |  |  |
| Just enough | Reference |  |  |  |  |  |
| More than adequate | 0.42 (0.24, 0.75) | 0.003 | 0.63 (0.36, 1.10) | 0.105 | 0.61 (0.35, 1.06) | 0.078 |
| Inadequate | 0.59 (0.26, 1.32) | 0.199 | 0.68 (0.30, 1.56) | 0.364 | 0.96 (0.43, 2.13) | 0.919 |
| **Residency year** |  |  |  |  |  |  |
| First-year | Reference |  |  |  |  |  |
| Second-year | 1.64 (0.83, 3.24) | 0.152 | 0.90 (0.45, 1.79) | 0.765 | 1.13 (0.59, 2.19) | 0.712 |
| Third-year | 0.70 (0.35, 1.40) | 0.311 | 1.16 (0.57, 2.34) | 0.686 | 0.80 (0.40, 1.59) | 0.526 |
| Fourth-year | 1.34 (0.56, 3.19) | 0.506 | 1.65 (0.69, 3.95) | 0.257 | 1.08 (0.46, 2.51) | 0.867 |
| **Original affiliation** |  |  |  |  |  |  |
| Ministry of Public Health | Reference |  |  |  |  |  |
| Ministry of Tertiary Education | 1.02 (0.60, 1.74) | 0.946 | 1.40 (0.82, 2.40) | 0.220 | 1.80 (1.07, 3.04) | 0.027 |
| **Departments** |  |  |  |  |  |  |
| Other departments | Reference |  |  |  |  |  |
| Major departments | 0.72 (0.42, 1.21) | 0.216 | 0.74 (0.44, 1.25) | 0.256 | 1.08 (0.64, 1.80) | 0.780 |
| **Total work hours,** per 1-hour increase | 1.05 (0.99, 1.11) | 0.121 | 1.00 (0.95, 1.05) | 0.996 | 0.97 (0.92, 1.03) | 0.353 |
| **Salary,** per 10,000 THB increase | 0.93 (0.77, 1.13) | 0.481 | 1.10 (0.91, 1.32) | 0.335 | 1.03 (0.85, 1.24) | 0.770 |
| **Model 3: Psychiatric determinants (*n* = 274)** | | | | | | |
| **Active psychiatric underlying disease** | 1.64 (0.45, 5.97) | 0.452 | 1.59 (0.57, 4.45) | 0.374 | 1.37 (0.49, 3.87) | 0.552 |
| **Had suicidal ideation in the last 12 months** | 6.59 (0.75, 57.54) | 0.088 | 3.17 (0.88, 11.47) | 0.079 | 1.16 (0.34, 3.97) | 0.807 |
| **Sleep duration** (hours/day during last week), per 1 hour increased | 0.93 (0.72, 1.20) | 0.577 | 0.92 (0.73, 1.16) | 0.471 | 1.07 (0.86, 1.33) | 0.525 |
| **Depressive symptoms**, moderate to high | 8.87 (4.28, 18.38) | <0.001 | 3.02 (1.75, 5.23) | <0.001 | 2.49 (1.44, 4.30) | 0.001 |
| **Model 4: Medical error, compared to having no medical error during the last 3 months (*n* = 288)** | | | | | | |
| **Severe medical error** | 1.08 (0.21, 5.49) | 0.922 | 1.58 (0.38, 6.50) | 0.525 | 2.20 (0.55, 8.73) | 0.262 |
| **Non-severe medical error** | 2.61 (0.90, 7.54) | 0.077 | 1.89 (0.75, 4.75) | 0.177 | 0.71 (0.29, 1.77) | 0.466 |
| **Medication prescription medical error** | 2.73 (1.23, 6.04) | 0.013 | 2.93 (1.42, 6.03) | 0.004 | 0.96 (0.48, 1.94) | 0.914 |
| **Laboratory order medical error** | 0.80 (0.31, 2.07) | 0.648 | 0.91 (0.37, 2.22) | 0.830 | 1.16 (0.49, 2.74) | 0.739 |
| **Confounder summary score (*n* = 238)** | | | | | | |
| Demographic | 2.04 (1.19, 3.51) | 0.010 | 2.31 (1.24, 4.32) | 0.008 | 2.82 (1.31, 6.08) | 0.008 |
| Working conditions | 2.38 (1.35, 4.21) | 0.003 | 3.39 (1.46, 7.87) | 0.005 | 2.55 (1.30, 5.00) | 0.006 |
| Psychiatric | 2.62 (1.82, 3.79) | <0.001 | 2.11 (1.32, 3.38) | 0.002 | 2.66 (1.41, 5.03) | 0.003 |
| Medical error | 2.92 (1.45, 5.87) | 0.003 | 2.31 (1.32, 4.05) | 0.003 | 1.27 (0.20, 8.00) | 0.802 |

aOR, Adjusted odds ratio.
